# Operationalizing One Health Employing Social-Ecological Systems Theory: Lessons From the Greater Mekong Sub-region

**DOI:** 10.3389/fpubh.2019.00085

**Published:** 2019-05-22

**Authors:** Bruce A. Wilcox, A. Alonso Aguirre, Nicole De Paula, Boripat Siriaroonrat, Pierre Echaubard

**Affiliations:** ^1^ASEAN Institute for Health Development, Mahidol University, Nakon Pathom, Thailand; ^2^Department of Environmental Science and Policy, George Mason University, Fairfax, VA, United States; ^3^Global Health Group International, Chiang Rai, Thailand; ^4^Department of Research and Conservation, Zoological Park Organization of Thailand, Bangkok, Thailand

**Keywords:** adaptive health management, complexity, One Health, sustainable development goals, transdisciplinarity, system thinking, ecology, adaptive cycle

## Abstract

The idea of the interdependency of the health of humans, animals, and ecosystems emerged from the interplay of theory and concepts from medicine, public health and ecology among leading thinkers in these fields during the last century. The rationale for One Health and its focus on the “human, animal, and environmental interface” stems from this legacy and points to transdisciplinary, ecological and complex systems approaches as central to One Health practice. Demonstration of One Health's efficacy, its wider adoption and continual improvement require explicit operational criteria and evaluation metrics on this basis. Social-Ecological Systems Theory with its unique conception of resilience (SESR) currently offers the most well-developed framework for understanding these approaches and development of performance standards. This paper describes operational criteria for One Health developed accordingly, including a protocol currently being tested for vector borne disease interventions. Wider adoption of One Health is most likely to occur as One Health practitioners gain an increasing familiarity with ecological and complex systems concepts in practice employing a transdisciplinary process. Two areas in which this inevitably will be required for significant further progress, and where the beginnings of a foundation for building upon exist, include: (1) Emerging and re-emerging zoonotic diseases, and (2) successful implementation of the United Nations (UN) Sustainable Development Goals (SDGs). The former includes the challenge of stemming the threat of new microbial pathogens, anti-microbial resistant variants of existing pathogens, as well as resurgence of malaria and other recalcitrant diseases. The applicability of SESR in this regard is illustrated with two case examples from the Greater Mekong Subregion, Avian Influenza (H5N1) and Liver Fluke (*Opisthorchis viverrini)*. Each is shown to represent a science and policy challenge suggestive of an avoidable social-ecological system pathology that similarly has challenged sustainable development. Thus, SESR framing arguably is highly applicable to the SDGs, which, to a large extent, require consideration of human-animal-environmental health linkages. Further elaboration of these One Health operational criteria and metrics could contribute to the achievement of many of the SDGs.

## Introduction

The interdependence of the health of humans, animals, and of ecosystems, along with the biological diversity they represent, is commonly described as the underlying tenet of One Health (1–4). As a research aim, understanding this interdependence is closely aligned with that articulated for sustainability science. Both are argued to require an integrated framework that includes concepts, principles and methods spanning multiple disciplines. This goes beyond the biomedical and ecological sciences to include social sciences and local knowledge (5). More generally, and especially in traditional ecological and health knowledge contexts, the broader concept of “local science” is arguably more appropriate (6).

This implies disciplinary integration, including, in particular, integration of elements of the biomedical and ecological sciences, as well as social sciences. Those typically trained as scientists and/or practitioners in biomedicine, public health or allied fields, including One Health proponents and workers, are not necessarily accustomed to cross-disciplinary integration. Few are simultaneously familiar with concepts and disciplinary jargons spanning human and veterinary medicine, environmental science, and ecology, let alone the social sciences. Yet, One Health, as a transdisciplinary, ecological and systems thinking endeavor (4, 7–9), additionally requires a working understanding of these concepts and associated methods. While notable progress recently has been made in this regard (4, 10–12), realizing One Health's full potential through its wider adoption, demonstration of its efficacy, and continual improvement demands explicit criteria and associated evaluation metrics (9, 13, 14).

Social-Ecological Systems Theory with its unique conception of resilience (SESR) as a complex adaptive system property, provides a framework that currently best meets this need for operationalizing One Health. Originally developed on the basis of studies of ecosystem dynamics, SESR has grown into a robust integrative, transdisciplinary approach that uniquely combines natural and social sciences perspectives. As a central postulate and heuristic tool SESR's adaptive cycle has proven widely applicable for understanding adaptation and sustainability across many types of systems (15). The fact that it is based on principles emerging from studies of ecosystem functioning applied to environmental management, including pest control, and sustainable resources management and development, makes it particularly applicable to One Health's focus on problems at the human-animal-environment interface, especially emerging zoonoses (16, 17).

In this paper, we review the key elements of SESR applicable to One Health and elaborate on recently developed explicit operational criteria including a protocol for One Health projects. We then illustrate with two examples where this has provided new insights into two high profile One Health problem areas related to the now endemic avian influenza (H5N1) and long endemic liver fluke (*Opisthorchis* spp.) transmission in the Greater Mekong Sub-region (GMS). Finally, we consider how these insights and the transdisciplinary frame offered by SESR render these and One Health challenges in general operationally inseparable from the region's sustainable development. These insights suggest how biomedicine, public health and ecology, while often in conflict, actually represent complementary perspectives and synergistic opportunities. Finally, we show how this approach could contribute to the achievement of many of the SDGs.

## Social-Ecological System and Resilience Theory

In a previous paper (18), we traced the history of how the cross-fertilization of ideas associated with ecology, health and sustainability led to the present interest in their linkages. The highlights of this scholarly and practical evolution include the development of SESR. Its application, the core elements of which are the novel conceptions, “resilience” and “adaptive management,” emerged from a re-conceptualization of ecosystems (conventionally defined as distinct from human systems) viewed as coupled human-natural systems as described in a series of landmark books (19–23).

“‘Resilience,” as represented in this body of work, is understood as an emergent property of these systems (i.e., managed forests, fisheries, rangelands, and natural ecosystems) (21–26), described as “complex adaptive systems” (CAS) (24, 25). This is distinct from the linear, equilibrium view of all living systems that remains relatively dominant in science. By contrast, CAS are far-from-equilibrium systems that exhibit non-linear dynamics and emergent properties (e.g., disease emergence). They are predictably unpredictable, despite human intentions. Moreover, our intervention programs become part of the system, a factor in its dynamics that further adds to their complexity and potential unpredictability. As human—animal—environment CAS are always changing (always have and always will), they are effectively moving targets from a management standpoint (26).

Along with the adaptive cycle as metaphor of these system's dynamics, the complex systems-based conception of resilience arguably provided the linchpin, which explains the science underlying sustainable development (27). This tying together of humans and nature, and subsequently the economics and ecology of biodiversity (28), represented critical break-throughs in our understanding of ecosystems as coupled human-natural systems and their transformations based on a synthesis of social and ecological system change theories (22). The term social-ecological systems subsequently was adopted as this body of theories and concepts serving as an integrative framework (23).

SESR represents a revolution of practical insights about how coupled human-natural systems learn, thus adapt to continuously changing internal and external conditions, based on extensive quantitative and qualitative model development and testing with real world cases (29). This includes models of knowledge-system integration, counting the critical roles of visioning and scenario building, leadership, agents and actors, social networks, and institutional change, all of which underlie adaptive capacity. SESR's applicability to zoonotic disease emergence was first pointed out over a decade ago (16, 17).

**Figure 1:**
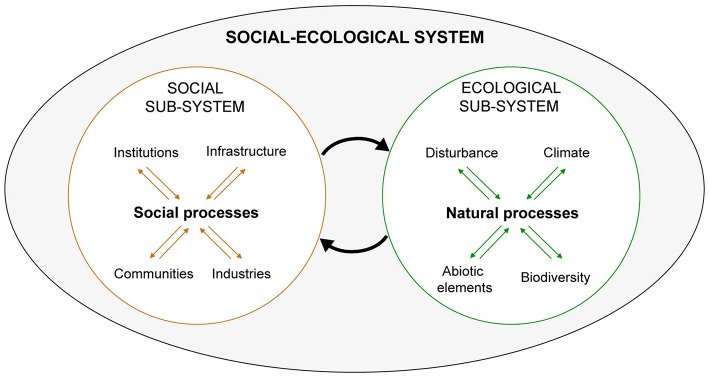
Graphical representation of a social-ecological system. The large oval represents an entire social-ecological system including its component social and ecological subsystems. The two large arrows in the middle represent interactions between them. For example, the arrow targeting the ecological sub-system represents human influences on nature. These are the outcome of processes influenced and/or driven by citizens, commercial interests, institutions (rules, regulations, customs), and the human-built infrastructure. They impact the ecological sub-system in numerous and often invisible ways mediated through ecosystem processes and functions, as a result of myriad abiotic and biotic interactions. The arrow targeting the social sub-system represents the outcome of all these factors. Adapted from Chapin et al. (30).

A social-ecological system can be envisioned as shown in Figure 1. The more commonly held conception of the human-nature relationship, at least previously in the environmental and ecological sciences, is that of humans impacting nature or vice versa, but generally not an ongoing co-adaptive (or maladaptive on the part of society) dynamic. SESR views the ecological and social subsystems as reciprocally linked by numerous interacting components as indicated by the two large arrows. For example, parasites and pathogens are an integral though largely invisible component of social-ecological systems and their dynamics, which nearly everywhere on the planet are undergoing dramatic, human-induced changes. How these alterations—most visible in terms of landscape change (e.g., deforestation, construction of dams and irrigation infrastructure, and cropland, and pastureland expansion) and less visibly through pesticides and other chemicals—affect parasite and pathogen diversity, abundance, and dynamics has been the subject of extensive research in, for example, the relatively new field of disease ecology (31).

The arrow in Figure 1 pointing from the social sub-system to the ecological sub-system represents this influence of human-related activities on natural systems. This arrow also represents the policy and management responses to the unintended “side-effects” of development, thus completing one feedback cycle. Such interventions typically are top-down and aimed at the control of ecosystem elements (e.g., vectors or parasites). While proving beneficial in the short-term they erode resilience in the long term. As widely documented in environmental and natural resources fields, such control attempts, including of pest populations, often result in a social-ecological system “pathology” (21, 22, 26). This involves a loss of resilience and sustainability with unintended consequences, which can include a return of the problem an agency originally sought to solve. Given its validation on the basis of extensive research and real world application, including continual refinement of understanding how to avoid these pathologies (15), SESR warrants serious consideration as the primary framing system for operationalizing One Health.

## The One Health Framing Problem and SESR

It is widely accepted that One Health requires systems thinking (4, 32), and specific One Health issues often are best addressed “on the ground” employing ecosystem approaches (1, 33). As alluded to above, SESR developed as an elaboration of thinking that originated with research on natural ecosystem behavior (34). The idea of adaptive management evolved from initial attempts to apply this thinking in the context of environmental assessment (19) and subsequently to natural resources management problems (20)—spanning issues such as the failed forest and crop pest control efforts and collapse of “scientifically managed” fisheries. Adaptive management became a core principle (and procedural component) of Ecosystem Management now widely adopted by natural resource management agencies worldwide. SESR subsequently emerged from the same school of thought, though Ecosystem Management can be seen as a special application (35). Thus, Ecosystem Management is SESR applied to areas of publicly and associated privately held lands with mapped legal and associated ecological boundaries (e.g., national parks and protected area complexes, eco-regions, or river basins).

The idea of the human-animal-environment nexus of One Health implies a similarly describable spatial context, whether a geographic or geopolitical region, and/or a place. Ideally, a target or “study” area's boundaries can be at least approximately delineated corresponding to an ecologically functional whole such as a watershed, or river basin. Or, this could be a contiguous habitat area supporting a particular set of ecological processes and interacting species spanning protected area's or state's boundaries (e.g., Serengeti ecosystem).

SESR application may not require a similar focus or depth of analysis of natural resources as typically conducted in Ecosystem Management. Instead, particularly in the context of zoonotic diseases it stresses coupled human-natural system's hierarchical organization (more accurately the embedded structure) and importance of considering cross-scale interactions in planning and management. This should include identification of key social (institutional), as well as relevant natural system components (e.g., vectors and their habitats), ecological interactions, and possible outcomes (e.g., the response of host-pathogen-environment complexes to interventions and vice versa).

While this of course requires the assemblage of appropriate kinds of disciplinary expertise (vector and host reservoir ecology in the above example), in our experience this is not a limiting factor in the uptake by One Health of SESR, and especially its intellectually challenging conception of resilience. Rather, the main difficulty seems to be that transdisciplinary research and integrative, holistic thinking challenge the conventional reductionist thinking and practice to which most of us are accustomed. Biomedical academic training and practice in clinical, laboratory, and even farm settings, tends to engrain a linear, reductionist way of thinking. This even holds for fieldwork including epidemiological studies and trials, which are purposely designed to “control” for real world complexity.

This default frame is often more than adequate, even powerful, including providing elegant mathematical explanations and associated interventions for infectious disease dynamics (e.g., the eradication of small pox and rinderpest and control of numerous infections that had previously plagued humans and livestock). However, the present global emerging zoonotic disease crisis demonstrates the reductionist biomedical frame is inadequate by itself for understanding and managing problems of host-pathogen-environment complexes (16, 36, 37).

## The Adaptive Cycle and Resilience

**Figure 2:**
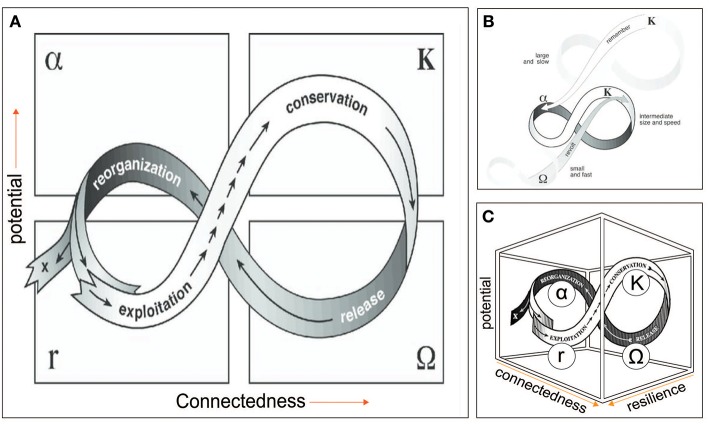
**(A)** Adaptive cycle. **(B)** Panarchy. Cross-scale linkages among adaptive cycles in a social-ecological system, in which successively smaller, faster cycles are embedded in larger, slower ones. **(C)** Three dimensional representation of the adaptive cycle. ***Potential*** represents resources in the form of stored capital available to effect change, which may include knowledge and financial, social, and natural capital; ***Connectedness*** refers to the flexibility or rigidity of controlling variables or processes in response to external variation; ***Resilience*** is the capacity of the system to absorb or withstand perturbations and other stressors such that it maintains its structure and functions [i.e., does not undergo regime change. Adapted from Gunderson and Holling (22)].

SESR's adaptive cycle metaphor (Figure 2) is central to understanding and navigating social-ecological systems as CAS's (24). The adaptive cycle explicates resilience and vice versa, while is also arguably key to understanding notions of health and sustainability in One Health. Although SESR originated with the study of natural ecosystems, viewing humans as outside agents, it was born from the realization that humans—acting as controllers of the natural system—can be thought of as both part of the system, and the problem (25). Even in the absence of human interference, ecosystems exhibit natural rhythms of change, the amplitude and frequency of which are determined by internal processes (e.g., ecological processes such as interspecific interactions) in response to past events. The discovery that these rhythms alternate periods of increasing organization and stasis with periods of reorganization and renewal was a significant break-through in the field of systems ecology, hence determining ecosystem productivity and resilience across scales (34).

The adaptive cycle (Figure 2A) is a metaphorical representation of the temporal and spatial patterning of these rhythms, which originated as a means of describing how conventional environmental management efforts involving ecosystems often fail over the long term (22). The adaptive cycles' four distinct stages are: (i) growth or exploitation *r*, (ii) conservation (*K*), (iii) collapse or release (Ω), and (iv) reorganization (α). It exhibits two major phases (or transitions). The first (fore loop), from *r* to *K*, is the slow, incremental phase of growth and accumulation. The second (the back loop) from Ω to α, is the rapid phase of reorganization leading to the system's renewal, or possibly a “flip” to a new stability domain. This also is referred to as a regime shift, which generally means a tipping point or threshold has been reached following which a social-ecological system “collapses” (38, 39).

These collapses can be triggered by politics, invasions, market shifts, or global climate change external to a system whose resilience at a particular scale has contracted due to states and dynamics at scales above and below (38). These collapses, described as panarchy (Figure 2C), were first discovered in studies of rangeland management systems. The adaptive cycle metaphor, including regime shift can be applied to relatively abrupt, irreversible agro-ecosystem transitions impacting livelihoods and human well-being (39). The adaptive cycle or the notion of social-ecological system pathologies in reference to landscape transitions has not yet been considered in the One Health literature to our knowledge. However, the applicability to One Health challenges is apparent as we describe here, using as an example the dramatic agro-ecosystem transformation underway in the Greater Mekong Sub-region (GMS). This mainly involves the widespread industrial intensification of agricultural production and food supply chain, with considerable but as yet not systematically investigated emerging zoonotic disease risks (40). This transformation, involving changing land use and land cover, increased chemical inputs including pesticides and anti-microbials, represents attempts to control a range of key variables including increased food and reduced pests and pathogen.

The initial phase of the adaptive cycle is driven by the “quest for increased economic growth.” This is the *Exploitation* phase during which the initial successes in each of these elements in terms of increased economic output reinforce the belief in the intensification approaches. Thus, increased investment and improvement (administrative, operational, organizational, technical, etc.), grows. The success breeds confidence and continues even when effectiveness of, for example, pesticides and antimicrobials begins to wane due to emergence of resistant strains.

The *Conservation* phase is illustrated here by the tendency to “double down” on the ongoing approach even as it becomes less effective, due to the system having become entrenched both in its thinking and mode of operation—reflecting a loss of flexibility, over-connectedness, and resulting fragility.

During the *Release* phase, a crisis stage is reached—i.e., the system clearly becomes unprofitable, as events such as major disease outbreaks, become increasingly costly for the controlling institutions. Prompted by revolutionaries within or outside of them pushing for change, the system enters the creative destruction phase. Assuming the system resilience remains sufficient, that is, sufficient adaptive capacity remains, and the system has not collapsed to a different state, irrevocably, the opportunity to reconfigure may exist. Thus, the system enters the reorganization phase and, hopefully, the result leads to desirable outcome.

A regional agroecosystem consists of many agroecosystem subtypes on the landscape scale, for instance, spanning small-scale traditional and small-holder systems to intensive large scale corporate industrialized systems. It can be envisioned how each has its own adaptive cycle whose dynamics operate on different time and space scales, including smaller, faster cycles being embedded in larger, slower ones (Figure 2B). The larger, slower cycles can constrain smaller, faster cycles, which the latter can disrupt and even cause a regime shift in which the social-ecological system is fundamentally altered.

As seen in the three-dimensional graph of the adaptive cycle (Figure 2C), resilience represents a third dimension that expands and shrinks through the cycle as slow variables change. It shrinks as the cycle moves toward K, and the system becomes more fragile, and expands abruptly when a cycle shifts into a “back loop” to reorganize for the initiation of a new cycle. “X” represents a regime shift whereby the system “collapses,” becoming a new system, functionally and structurally. The back loop, Ω phase, is a period in which novelty and experimentation is needed and possible, given a decline in connectedness (e.g., as institutional rigidity or inflexibility diminish) and increase in resilience. It constitutes an opportunity for “revolt,” a cross-scale phenomenon precipitated by fast, small variables.

As recently pointed out (15), a resilient system may successfully navigate itself through each of the phases and into new regime that satisfies societal goals. In general, however, successful navigation (an indication of resilience) suggests the capacity to recognize barriers, critical thresholds and principles associated with this front loop that can trap a system—resulting in a pathology. System features, allowing escape from these traps, have been provisionally described (15)—representing adaptive management.

## Case Examples From the Greater Mekong Subregion

**Figure 3:**
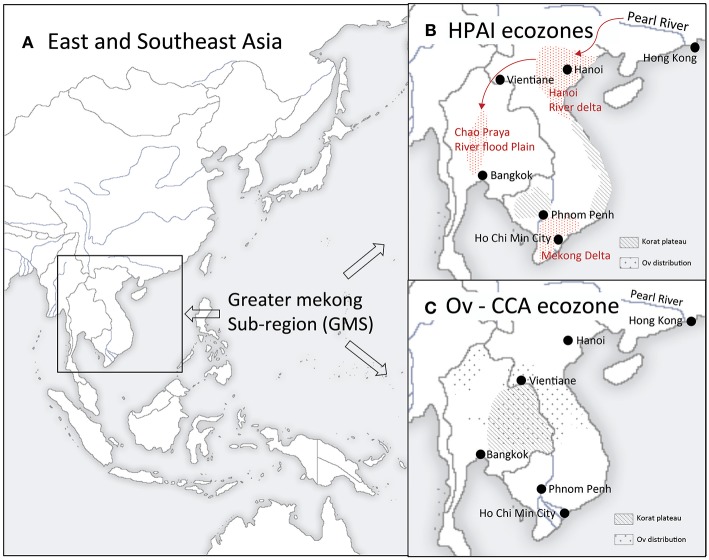
Greater Mekong Subregion and ecozones of the case examples. **(A)** East and Southeast Asia. The Greater Mekong Subregion includes six nations (Cambodia, China, Laos, Myanmar, Thailand, and Vietnam) whose national boundaires overlap the hydrographic boundaries of the Mekong River Basin. **(B)** HPAI ecozones. Red stippled areas indicate the distribution of Highly Pathogen Avian Influenza (H5N1) cases during the 2003-4 emergence/pandemic. These three perennial flood zones of Thailand (Chao Praya River flood plain) and Vietnam (Red River and Mekong Deltas) are centers of concentration of domestic poultry production as well of wild water birds including the primary natural host reservoirs for avian influenza viruses, dabbling ducks. Chickens and domesticated duck production, trade networks, and wet markets grew explosively beginning in the 1980's in response to increased export market demand. This represented a social-ecological system transformation and ultimately a regime shift from once the first H5N1 virus variant invaded Vietnam's Red River Delta from the Pearl River Basin or elsewhere in China. The Red River Delta may have provide the key stepping stone to southern Vietnam's Mekong Delta and Thailand's Chao Prya River Basin. Following these first epidemic waves outbreaks were brought under control in the Chao Prya, but not in Vietnam where HPAI outbreaks continue to occur. **(C)** Liver fluke-Liver Cancer ecozones. *Opisthorchis viverrini* infection in humans is wide-spread throughout its geographic distribution. Yet its epidemiological characteristics in the Korat Plateau are distinct, reflecting its unique culture and environment. The Plateau historically had been sparsely populated (by human's and likely the liver fluke as well) but grew exponentially since WWII while dams and irrigation systems transformed the Plateau's social-ecology including expanding favorable aquatic habitat for *Bythnia* snails, *O. viverrini'*s first intermediate host. The Plateau's population (~ 25 million people today) of predominantly Lao dialect-speaking, a rice-fish culture for the second intermediate host, is a staple food. As is observed when they relocate today within Thailand, along with this food cultural practice, they would have carried the fluke with them from their area of origin in Southern China as they migrated southward during the last millenium.

This paper offers two case studies on One Health efforts employing SESR based on our work in the Greater Mekong Subregion (GMS) (Figure 3). The first is related to food production intensification found to represent a substantial range of health threats realized most dramatically with the emergence of the H5N1 strain of highly pathogenic avian influenza (HPAI) (40). An interdisciplinary research effort supported by the US National Science Foundation Coupled-Human Natural Systems Program sought to apply an SESR frame to better understand the causes of HPAI emergence (41).

The principal findings of this effort (42) are summarized as follows. The initial outbreak in Hong Kong, when H5N1 was first isolated, was optimistical but mistakenly thought to have been successfully eliminated by massive poultry culling in 1998 (43). However, experts and government authorities either ignored or otherwise were oblivious to the change (large, slow variable in SESR parlance) taking place regionally. This consisted of growth of industrial scale poultry operations in Guangdong, China, and particularly the transformation of the poultry production landscape taking place in geographically adjacent Vietnam. This included dramatic changes in breed composition and flock size, and the expansion of this intensification across Vietnam (linking the north with the south of the country), as well as into Cambodia, Laos, and Thailand via the Mekong corridor in the south (Figure 3B).

These changes were pushed by global forces, driven by the economic opportunity presented by the growing export market. This historically unprecedented production intensification was not accompanied by similarly intensified biosecurity measures. The operations in China, which included breeding facilities with up to a million birds, constituted a crucible of genetic innovation (a small, fast variable). Among the untold new microbial variants generated, H5N1 variants sporadically and unpredictably spilled south over the China-Vietnam border via local trade networks and migrating ducks (another small, fast variable).

This might have been of limited consequence, as it was geographically speaking with the Hong Kong outbreak, where this agro-ecosystem's insularity helped prevent H5N1's escape to the south and ultimately globally. But it was the ongoing transformation, which is retrospectively even observable via satellite and on the ground (41, 42). This represented “an accident waiting to happen,” which did as evidenced by the explosive epidemic waves of 2003-4 initiated in Vietnam with H5N1 spreading to 60 countries. This fits the classic case of “surprise” in SESR jargon defined as a cognitive disagreement with expectations based on the responsible social institution's failure to recognize signs indicating the system's increasing fragility (22).

HPAI is now endemic throughout the Mekong Region (as well as in parts of insular Southeast Asia and Egypt), as part of a new social-ecological regime. This new regime effectively represents a regional agro-ecosystem distinct from that previously, structurally and functionally, including a number of less desirable social, economic, political, environmental, conservation and public health features. This includes most prominently a far less diversified poultry production sector now dominated by large agribusinesses, increased dependence on agrichemicals, drugs, and vaccines. In addition, outbreaks threatening wildlife including in protected areas have been recurring (Dr. Paisin Lekchareon, personal comm). Transboundary movements involving product supply chains for multi-country operations may play an important role in AI virus transmission ecology along with human movement.

The emergence of HPAI (H1N1) demonstrates SESR's value applied as a retrospective method of analysis in the elucidation of the previously unexplained details of its pandemic emergence. It also offers numerous insights related to the HPAI and newly emerging avian influenza strains in the GMS as a One Health problem area that is intertwined with the multitude of issues related to the intersection of environment, conservation, development, and public health—thus, inevitably sustainable development. It remains to be seen whether the integrative perspective and problem-solving approach SESR offers will be considered by regional and/or national bodies in the GMS.

Our second case example has prompted actions among GMS countries toward implementing changes in interventions based on SESR. In this case, we (BW and PE) were recruited to assist with applying an ecosystem approach to the problem of Liver Fluke (*Opisthorchis viverrini*) infection as a putative cause of the relatively high incidence of liver cancer (cholangiocarcinoma) in Northeastern Thailand, “Isaan” encompassing the Korat Plateau of the Lower Mekong basin (Figure 3C). This work included designing and conducting research aimed at filling gaps in understanding environmental and ecological aspects of the parasite's transmission. More generally this resulted in broadening the understanding of the social and ecological dimensions of liver fluke transmission and its role in disease (44, 45).

In addition to resulting in a rethinking of the Ov-CCA problem (46–50), this effort drew attention to a deficiency in the larger program with which this research was affiliated: the absence of explicit criteria and procedures for applying “ecosystem approach to health” (51). Among the outcomes prompted by this is the recognition of complex systems thinking including transdisciplinary methods required by the ecosystem approach (52).

Despite a diversity of perspectives on the Ov-CCA problem—held by stakeholders with very different perceptions, values, objectives and even social standing (e.g., university professors, public health practitioners, social anthropologists, government representatives and villagers)—a biomedical research frame (consistent with the biomedical model) as the basis of the design of public health interventions had been accepted by default to the exclusion of any others. This included that held by “risk groups” themselves, mainly villagers and farmers, who do not perceive eating fermented fish as a particularly high-risk behavior. Rather, they view it not only as normal behavior, but as an integral part of their daily life. The beliefs and practices related to preparing, sharing and eating fermented fish dishes, along with rice cultivation and consumption are inseparable from their cultural identity, their local natural capital, and social capital as evidenced by fish dish sharing networks in villages (49). An exclusively biomedical model-driven research and intervention agenda prevailed for decades despite evidence of its limited capacity to effect a decrease in infection prevalence or CCA incidence. Added to this is this agenda's potential dangers with regard to other health and well-being dimensions that are inadvertently affected by targeted liver fluke-CCA interventions (45, 47).

In fact, evidence from existing data or studies, as well as that from new results from the recently added social and ecological components, suggests villagers perceptions, or the “lay model,” is apparently no less valid than the biomedical model. At least four different models of *Ov* and health connection are recognizable (47), none of which, including the biomedical model, are completely wrong, but just incomplete. In the final analysis it can be seen how the *Ov*-CAA problem is a “moving target” and the targeted attempt to control, top down, a social-ecological variable, prevalence of infection through consumption of traditional fish dishes, represents a “disease control pathology” in the classical SESR sense (16). The Isaan-Lao cultural and natural ecology (livelihoods, environmental exposures, and human and land health profiles, i.e., social-ecological system) of the Korat Plateau effectively have been undergoing a regime shift. The multitude of interacting factors likely responsible for the region's relatively high CCA incidence as recently demonstrated (46), almost certainly also have been shifting as well. The appreciation of this offered by SESR has stimulated the beginning of a “revolt” akin to the panarchy (Figure 2B).

## Transdisciplinary Process for Building Adaptive Capacity

**Figure 4:**
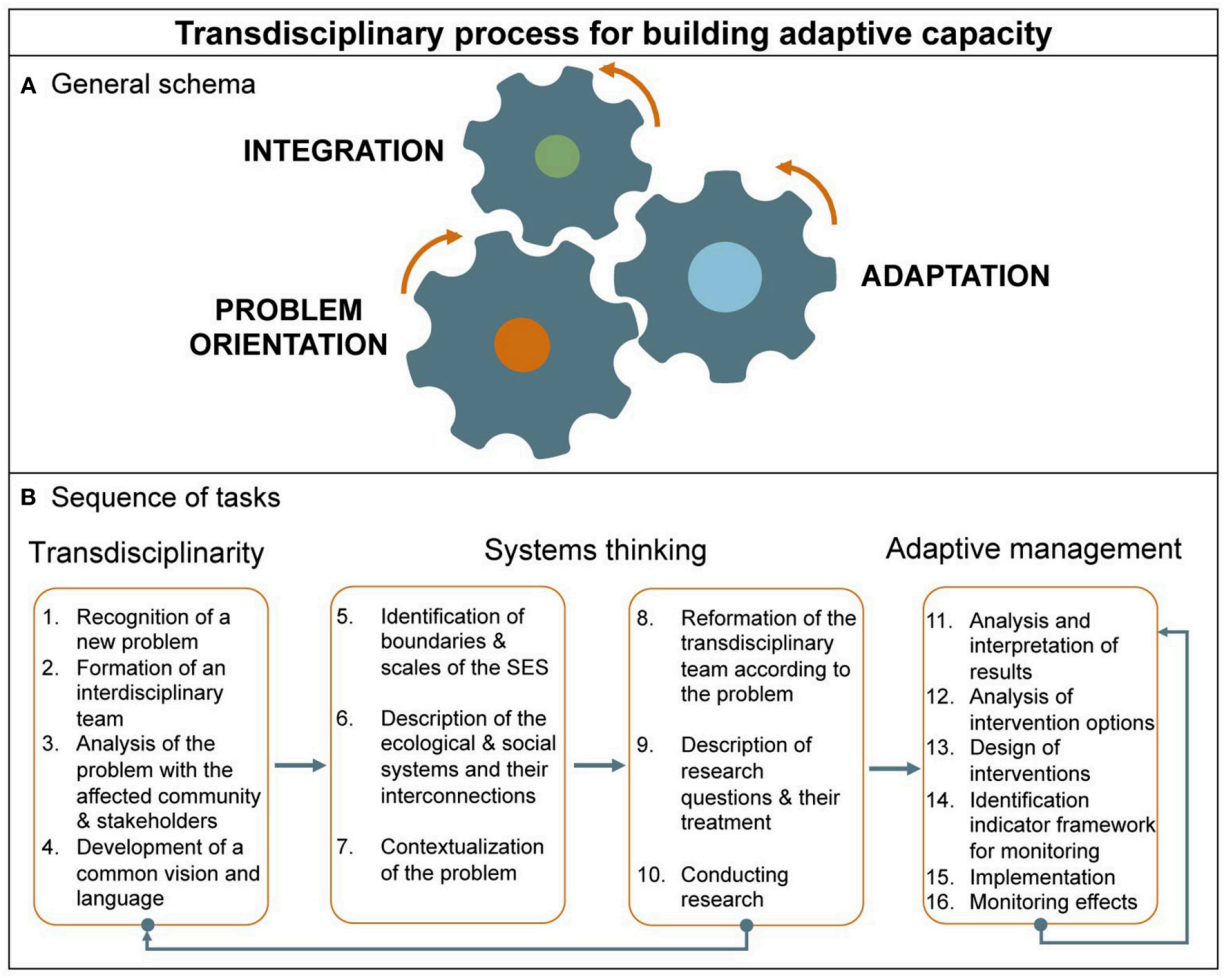
Transdisciplinary process for building adaptive capacity. **(A)** Transdisciplinarity can be envisioned as a process involving three mutually reinforcing, and overlapping activities: problem orientation, integration and adaptation (as defined in the text). **(B)** This process can be further broken down into a sequence of specific actions representing a protocol against progress including conformance to One Health as a transdisciplinary, ecosystem approach could be measured, when combined with the criteria described in Table 1 [adapted from Richter et al. (53)].

Perhaps the most important lesson learned from these case examples is how defaulting on one perspective, or model, resulted in an only partial understanding of the problem—thus only partial or temporary solutions. This invokes transdisciplinarity in which a ongoing process of “problem orientation”—that is, sharing of different understandings, reflecting and even negotiating around a definition of “the problem”—is requisite from the very beginning. As illustrated in Figure 4A, this drives, at least initially, integration, and ultimately adaptation, although as a transdisciplinary process develops each feeds back on the others. Figure 4B decomposes this simplified description of these three interrelated processes into a suggested stepwise protocol, based on an analysis of criteria along a continuum of increasing comprehensiveness (from weak to strong transdisciplinarity, (54) and increasing resilience as further described in Table 1.

**Table 1:** Dimension of resilience containing sequential criteria of progressing comprehensiveness.

**Resilience**	**Weakest**	**Weak**	**Intermediate**	**Strong**	**Strongest**
Transdisciplinarity	Integration	Composition	Differentiation	Collaboration	Value creation
Community participation	Representation	Involvement	Partnership	Empowerment	Autonomy
System thinking	Scoping	System description	Problem analysis	Mitigation	Adaptiveness

*Resilience at multi-stakeholder group level is expected to be strongest when common values and understandings, as well as aims, are emerging from iterative negotiations. Adding onto that, when community stakeholders are intended beneficiaries, community members need to be co-designers at the onset of a research and development project while being provided with the tools and forums for inclusion and expression. A strengthening element leading to innovation and adaptive capacity is system thinking and the capacity to identify relevant entities and influences accoss scales. Adapted from (53)*.

The transdisciplinary system's purpose is the cooperative generation of knowledge to solve, mitigate, or prevent a complex societal problem—such as involving diseases and environmental degradation—as distinct from academically defined problems solvable though conventional disciplinary research. In “real life,” academics and social actors often disagree on problem's causes, consequences, and problem-solving strategies. Transdisciplinarity involves grasping complexity, accounting for the diversity of perceptions, linking abstract and case-specific knowledge, and development of knowledge and practices for the common good. The manner and the extent to which different parts of “a whole” interact need to be agreed upon and reevaluated throughout as a means of encompassing the problem. In this regard, it is useful to designate a phase devoted to the task of drawing on systems thinking (4). Typically, this involves identifying (literally drawing a schemata, mental maps, or actual geographic maps) the system's boundaries and the social and ecological components and their interconnections.

The adaptive cycle metaphor not only describes social-ecological system pathologies and how they lead to management failures, but also successes, employing adaptive management, described by cyclic phases consisting of learning, describing, predicting, and doing [Figure 2; (55)]. These tasks are a prerequisite for a program of adaptive management that includes consideration of intervention options accounting for feasibility, development of a system of metrics for monitoring interventions' impacts, reconsideration and redesign of interventions, evaluation, and so on, as a cycle (Figure 4B).

As has been cogently explained by its originators (21), adaptive management is an inductive approach, relying on comparative studies, ecological theories and observations, and the design of planned interventions in nature with an understanding of how, in turn, humans are likely to respond. Its aim is to identify uncertainties, test hypotheses about them, thereby employing interventions (management) as a tool to change the system. In the process, it is learned how to match the human and natural dynamics across scales, thus insure greater resilience: That is, the “enhanced capacity to deal with change and surprise, including avoiding shifts to undesirable stability domains, while providing flexibility and opportunity in a rapidly changing and human dominated planet” (22).

Finally, a major challenge for One Health, only briefly touched on above, but also apparent from SESR, is the role of the political economy, as well as systems ecology. One Health approaches arguably should include consideration, if not investigation, of the links between “macro-structural” contexts affecting local agro-ecosystem/agro-economic circumstances. For example, this includes those ultimately responsible for the evolution and emergence of highly pathogenic avian influenza H5N1 (56), which became and remains an ongoing human-domestic animal-wildlife problem globally. This is consistent with ecosystem thinking and SESR in particular, which points to the importance of considering social-ecological systems' hierarchical organization and associated cross-scale institutional, as well as ecological dynamics involved in zoonotic disease emergence (57). Ultimately “adaptive health governance,” involving coordination among disciplinary, sectorial and public domains, is required (58).

## One Health: Informing the Implementation of the UN Sustainable Development Goals

The lessons from the past several decades since the idea of sustainable development was introduced together with more recent science-based global policy frameworks, such as the Millennium Development Goals (MDG), suggest the need for a more in-depth understanding of human-nature systems, as offered by SESR. Shortcomings of the Millennium Ecosystem Assessment, a key component underlying the MDG strategy, reflected a failure to account for how both ecosystems and health systems are complex adaptive systems (18, 59). One Health's transdisciplinary, ecosystem approach imperative, along with SESR's operational relevance to sustainable development, presents a current and unique opportunity for bridging science-policy gaps. This would allow an improved synergy between practitioners, and environmental-health policies.

The health-sustainable development linkage has a strained policy history. Health was addressed only very obliquely by the World Commission on Environment and Development (WCED) in the lead up to the historic 1992 UN Earth Summit in Rio de Janeiro, Brazil. In the historic and widely read report titled “Our Common Future,” also known as the Brundtland Report, WCED codified and was the first to explicitly define sustainable development. This framed the 1992 Rio Earth Summit and its key outcomes, international treaties on climate, biodiversity and desertification, as well as non-binding Agenda 21, which was dominated by environmental and conservation perspectives of health. For example, the “control of communicable diseases” was mentioned without further elaboration (60). The World Health Organization (WHO), the UN's chief health agency, was largely left out of the policy formulations.

In the years following, WHO's administrators, being keenly aware of having played “back seat” role at best, developed a number of programs aimed at remedying this by focusing on environmental health. Yet, many countries did not officially affirm the linkages between public health and the health of the environment (60). This changed somewhat at the 2002 Summit on Sustainable Development and more recently with the UN Sustainable Development Goals (SDGs), although major gaps remained. This slow progress in recognizing the synergies between health and the environment continues to be a reflection of the epistemological gaps separating the health sciences from the fields of environmental science, economics, and international development.

The SDGs, seen as a response to the limitations of the Millennium Development Goals (MDGs), are an opportunity to further integrate health, environment and development. Several UN agencies have noted the need and opportunity the promulgation of SDGs offers to One Health, from the standpoint of the synergistic possibilities among sectors. Although only three of the SDGs explicitly mention health, most (if not all 17) indirectly can be linked to the “principles” supporting the One Health approach and having a direct or indirect impact on the main cornerstones of sustainable development as they relate to zoonotic and vector-borne diseases and extend to salutary factors (Table 2). The authors point out the SGDs' requirement of an integrated response is similar to what recent programs addressing neglected tropical diseases (NTDs) have defined (61). The vast majority of NTDs are zoonoses. Thus, their assessment is applicable to the One Health tenets.

**Table 2:** United Nations Sustainable Development Goals (SGDs) from a One Health Perspective as they relate to zoonotic and vectorborne diseases.

**SDG**	**Brief description**	**Application to one health example**
1	End poverty in all its forms everywhere	Addressing challenges at the interface of human, animal and ecosystem health are inextricably and reciprocally linked to poverty. Zoonotic infections exacerbate poverty and vice versa.
2	End hunger, achieve food security, improve nutrition and promote sustainable agriculture	Chronic parasitic infections often exacerbate caloric and nutritional deficits in people and animals, not only affecting the productivity of infected farmers but livestock production as well.
3	Ensure healthy lives and promote well-being for all	One health interventions are particularly relevant in terms of reaching people with limited access to health systems in rural areas, as well as other development-related goals addressed by Agenda 2030.
4	Ensure inclusive and equitable quality education and promote lifelong learning opportunities for all	Zoonotic diseases can stigmatize affected students, reduce attendance and school performance. Also, school based health education programs including targeting control of specific diseases (e.g., arbovirus vector community-based control) can be highly effective.
5	Achieve gender equality and empower all women and girls	Women are disproportionately affected by poverty, illiteracy, lack of education, land ownership and political voice, and access to health care. They also have greater exposure to a number of diseases through their domestic and other work roles.
6	Ensure access to water and sanitation for all	Water, sanitation, and hygiene activities associated with prevention and control of zoonoses require integration with a range of cross-sectoral activities aimed at interrupting transmission cycles of many zoonotic and vector borne diseases.
7	Ensure access to affordable, reliable, sustainable and modern energy for all	Construction of hydroelectric dams often alter local ecological conditions favoring vectors, while vector control and organic waste management as a sanitary measure via biogas systems for example align with sustainable energy development.
8	Promote inclusive and sustainable economic growth, employment and decent work for all	Zoonotic infections represent a large burden to health care systems and negatively impact economic productivity, which can be significantly mitigated by zoonotic disease prevention and control.
9	Build resilient infrastructure, promote sustainable industrialization, and foster innovation	Interventions targeting neglected populations require development of transport and storage infrastructure as well as clinics for the provision of health services including distribution of donated medicines.
10	Reduce inequality within and among countries	Interventions targeting the most disadvantaged and marginalized populations whose disease prevalence typically is highest will contribute to reducing socio-economic disparity.
11	Make cities inclusive, safe, resilient, and sustainable	Mosquito and other disease vectors adapted to urban habitats continue to proliferate with urbanization. Integrated, community based vector control interventions will make cities more livable and resilient.
12	Ensure sustainable consumption and production patterns	Waste management aimed at sustainable use, reuse, and recycling simultaneously can address control of nuisance or disease vector mosquitos and other threats including from pesticide exposures.
13	Take urgent action to combat climate change and its impacts	Though currently largely unpredictable, changes in temperature, rainfall and relative humidity associated with global environmental change affect the dynamics and spread of disease vector populations. One Health oriented research on these linkages will contribute to reducing these potential risks.
14	Conserve and sustainably use the oceans, seas and marine resources	Coastal populations throughout tropical developing regions, where Aedes mosquitoes are ubiquitous, are thus even vulnerable to the negative socioeconomic consequences of marine resource degradation, as impoverishment reduces prospects for vector control and avoiding more severe disease outcomes upon infection of high zoonotic disease prevalence. Thus improved sustainable management of marine ecosystems will positively impact zoonotic disease control.
15	Sustainably manage forests, combat desertification, halt and reverse land degradation, halt biodiversity loss	Zoonotic disease emergence is known to be facilitated by deforestation, the disturbance and degradation of natural and semi-natural habitats, and in particular biodiversity loss. This includes that of natural ecosystems as well as agroecosystems (agrobiodiversity) including plants and animals cultivated and domesticated by farmers over millennia.
16	Promote just, peaceful and inclusive societies	Zoonotic disease epidemics frequently are associated with political and armed conflict. Interventions aimed at affected civilian populations at times and places can be a tool to promote peace.
17	Revitalize the global partnership for sustainable development	Integration of all the above requires new as well as the strengthening of existing partnerships among a wide breadth of interests spanning private and public organizations and agencies in the spirit of transdisciplinarity.

## Conclusions

In order to operationalize One Health, there is a need for: a “proof of concept” incorporating environmental and ecosystem factors (13); integration and systems thinking (4, 12); standardized evaluation metrics (14); and a framework incorporating the broad social, as well as biological aspects.

Unprecedented human impacts and transformation of Earth's ecosystems have become the most pressing global threat to human health and well-being. Knowledge integration and working collaboration among scientists on the one hand and among multiple sectors, NGOs, local communities, and policy makers on the other is imperative. Thus, rethinking how we approach health in relation to our environment, drawing on a One Health approach understood as a transdisciplinary and ecosystem-based endeavor, particularly in light of new insights offered by SESR, is timely.

The United Nations 2030 Agenda for Sustainable Development, signed in 2015, is perhaps the most significant effort yet undertaken to address the above threat. The nations of our planet and their leaders have universally agreed to this new and more comprehensive development road map. This requires integration of markedly different perspectives, perceptions, values and normative conventions representative of the fields spanning the 17 goals. It is certain that all 17 SDGs can only effectively be implemented if public policies reflect a truly integrated global policy agenda. This can only be achieved through an understanding of health as seen through a transdisciplinary, ecosystem-oriented lens.

## Author Contributions

BW and PE developed the rationale and the overall orientation of the ideas. AAA, BW, ND, BS, and PE wrote the manuscript. All the authors read and approved the final manuscript.

### Conflict of Interest Statement

The authors declare that the research was conducted in the absence of any commercial or financial relationships that could be construed as a potential conflict of interest.
